# Effect of Selected Cooking Ingredients for Nixtamalization on the Reduction of *Fusarium* Mycotoxins in Maize and Sorghum

**DOI:** 10.3390/toxins13010027

**Published:** 2021-01-04

**Authors:** Julianah Olayemi Odukoya, Sarah De Saeger, Marthe De Boevre, Gabriel Olaniran Adegoke, Kris Audenaert, Siska Croubels, Gunther Antonissen, Karel Vermeulen, Sefater Gbashi, Patrick Berka Njobeh

**Affiliations:** 1 Department of Biotechnology and Food Technology, Faculty of Science, University of Johannesburg, Doornfontein Campus, P.O. Box 17011, Gauteng 2028, South Africa; julianahodukoya@gmail.com (J.O.O.); sgbashi@uj.ac.za (S.G.); 2 Centre of Excellence in Mycotoxicology and Public Health, Department of Bioanalysis, Faculty of Pharmaceutical Sciences, Ghent University, 9000 Ghent, Belgium; marthe.deboevre@ugent.be; 3 Department of Food Science and Technology, Kwara State University, Malete P.M.B. 1530, Kwara State, Nigeria; 4 Department of Food Technology, Faculty of Technology, University of Ibadan, Ibadan 200284, Nigeria; goadegoke@yahoo.com; 5 Department of Plants and Crops, Faculty of Bioscience Engineering, Ghent University, 9000 Ghent, Belgium; Kris.Audenaert@ugent.be; 6 Department of Pharmacology, Toxicology and Biochemistry, Faculty of Veterinary Medicine, Ghent University, Salisburylaan 133, 9820 Merelbeke, Belgium; Siska.Croubels@ugent.be (S.C.); Gunther.Antonissen@ugent.be (G.A.); 7 Department of Pathology, Bacteriology and Avian Diseases, Faculty of Veterinary Medicine, Ghent University, Salisburylaan 133, 9820 Merelbeke, Belgium; 8 Department of Data Analysis and Mathematical Modelling, Faculty of Bioscience Engineering, Ghent University, 9000 Ghent, Belgium; KarelB.Vermeulen@ugent.be

**Keywords:** cooking ingredients, food safety, *Fusarium* mycotoxins, LC-MS/MS, maize, nixtamalization, sorghum, food processing

## Abstract

Although previous studies have reported the use of nixtamalization for mycotoxins reduction in maize, the efficacy of calcium hydroxide and other nixtamalization cooking ingredients for mycotoxin reduction/decontamination in sorghum and other cereals still need to be determined. The current study investigated the effect of five nixtamalization cooking ingredients (wood ashes, calcium hydroxide, sodium hydroxide, potassium hydroxide, and calcium chloride) on the reduction of *Fusarium* mycotoxins in artificially contaminated maize and sorghum using liquid chromatography-tandem mass spectrometry. All tested cooking ingredients effectively reduced levels of mycotoxins in the contaminated samples with reduction initiated immediately after the washing step. Except for the calcium chloride *nixtamal*, levels of fumonisin B_1_, B_2,_ and B_3_ in the processed sorghum *nixtamal* samples were below the limit of detection. Meanwhile, the lowest pH values were obtained from the maize (4.84; 4.99), as well as sorghum (4.83; 4.81) *nejayote* and *nixtamal* samples obtained via calcium chloride treatment. Overall, the results revealed that the tested cooking ingredients were effective in reducing the target mycotoxins. In addition, it pointed out the potential of calcium chloride, though with reduced effectiveness, as a possible greener alternative cooking ingredient (ecological nixtamalization) when there are environmental concerns caused by alkaline *nejayote*.

## 1. Introduction

Mycotoxins are secondary fungal metabolites that often contaminate agricultural commodities in the field or during storage [1,2,3,4]. The fungal species producing these mycotoxins are particular members of the *Fusarium*, *Aspergillus*, *Penicillium,* and *Alternaria* genera that pose serious health-related challenges in humans as a result of their toxigenic characteristics [5,6]. *Fusarium* mycotoxins, such as fumonisin B_1_ (FB_1_), fumonisin B_2_ (FB_2_), fumonisin B_3_ (FB_3_), deoxynivalenol (DON), 3-acetyl deoxynivalenol (3-ADON), 15-acetyl deoxynivalenol (15-ADON), nivalenol (NIV), and zearalenone (ZEN), among others [7], are of serious concern as they cause economic losses, trade barrier and human health problems like anorexia, diarrhea, cancer, and immunosuppression [1,8].

Nixtamalization is a major processing procedure employed in the preparation of some maize-based products including masa, tortillas, tortilla chips, and pozole [9]. It is a special food-processing technique in that it can cause several physicochemical modifications to maize kernels, contribute to flavor and affect mycotoxins whereby the latter (i.e., mycotoxins) might be degraded, modified, or released/bound in food [10].

In line with Escalante-Aburto [11], this processing technique (nixtamalization) can be grouped into three categories, namely, *classic*, *traditional* (involving the commonly used calcium hydroxide and other nixtamalization cooking ingredients), and *alternative* technologies. The *classic* nixtamalization, used in Mexico and Central America, involves the application of wood ashes [12] substituted with lime in *traditional* nixtamalization to ensure higher levels of pericarp removal [11]. Several authors, including Figueroa et al. [13], Ramírez-Jiménez et al. [14], Ramírez-Araujo et al. [15], and Enríquez-Castro et al. [16] have reported the use of lime as a *traditional* nixtamalization process, which according to Schaarschmidt and Fauhl-Hassek [10] is an effective as well as a promising technique for reducing mycotoxins while enhancing nutrient availability in maize. Nonetheless, the use of calcium hydroxide has been linked with environmental pollution issues arising from the disposal of its by-product, *nejayote* (wastewater), which usually has a high pH [17]. Other alkaline ingredients for nixtamalization, such as sodium hydroxide and potassium hydroxide, have also been used in the food industry [12]. For instance, sodium hydroxide is used to pre-cook grains for pozole preparation [12,18]. Based on environmental pollution issues caused by conventional nixtamalization, ecological nixtamalization, an *alternative* technology involving the use of calcium salts such as calcium chloride, calcium sulfate, and calcium carbonate, has been proposed with a reduced pH of the residual solution as a key advantage [11].

Although cereals like maize and sorghum contain essential nutrients [19], they are susceptible to natural contamination by toxigenic fungi accompanied by the production of mycotoxins [20,21]. This study, a MycoSafe-South project, thus, investigated the effect of some selected cooking ingredients for nixtamalization (wood ashes, calcium hydroxide, sodium hydroxide, potassium hydroxide, and calcium chloride) on the reduction of *Fusarium* mycotoxins in artificially-contaminated maize and sorghum grains during nixtamalization. The study is of immense importance as most investigations involving the use of calcium hydroxide during nixtamalization for mycotoxins reduction focused solely on maize with none on sorghum. Besides, no study has been carried out to establish the effect of wood ashes, sodium hydroxide, potassium hydroxide, and calcium chloride on the reduction of *Fusarium* mycotoxins in maize and sorghum during nixtamalization.

## 2. Results and Discussion

### 2.1. Influence of Different Nixtamalization Cooking Ingredients on Fusarium Mycotoxins during Nixtamalization of Maize

In this study, the effect of nixtamalization processing steps on *Fusarium* mycotoxins during the production of nixtamalized maize and sorghum was investigated. The substrate (maize and sorghum grains) used as starting materials were tested and had *Fusarium* mycotoxins concentrations below the limit of detection (LOD) (Table 1). After artificial inoculation with toxigenic *Fusarium verticillioides*, six toxins (FB_1_, FB_2_, FB_3_, DON, NIV, and ZEN; Table 2) were detected in the maize samples confirming this cereal as an ideal medium for the production of the targeted *Fusarium* mycotoxins [22].

According to Karlovsky et al. [23], water-soluble mycotoxins, including DON [24], may be removed by washing the outer layer of grains, while Humpf and Voss [25] noted that the water-solubility characteristics of fumonisin mycotoxins such as FB_1_, FB_2,_ and FB_3_ determine the rate at which they may be affected by grain washing. In this experiment, washing of the inoculated maize samples led to a significant reduction (*p* < 0.05) in the levels of the detected mycotoxins except for ZEN, where there was no statistically significant difference (*p* = 0.74) in the mycotoxin content before and after washing (Table 2). NIV was completely depleted at this stage, which reflects its high solubility in water as pointed out by Karlovsky et al. [23]. Similarly, the low solubility of ZEN in water also explains the non-significant difference (*p* > 0.05) of this mycotoxin in the washed and unwashed samples. Generally, results of the mycotoxin levels obtained after washing are in agreement with the predicted solubility values for FB_1_ and FB_2_ (>20,000 mg/L), DON (36,000 mg/L), NIV (64,600 mg/L), and ZEN (117 mg/L) previously established by Karlovsky et al. [22]. In addition, the total fumonisin reduction by washing (74%) was found to be in line with that obtained in previous studies by Humpf and Voss [26] (73%), Matumba et al. [27], as well as Shetty and Bhat [28] (74%).

**Figure 1:**
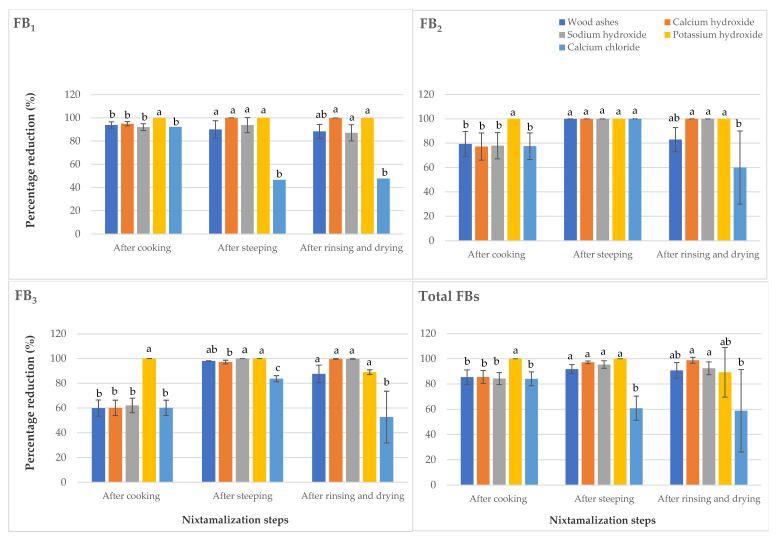
Reduction of fumonisins during the processing of nixtamalized maize samples using different nixtamalization cooking ingredients. Values are the means of five replicates ± standard error. Means followed by different letters are significantly different (*p* < 0.05) according to Tukey *post-hoc* test.

**Figure 2:**
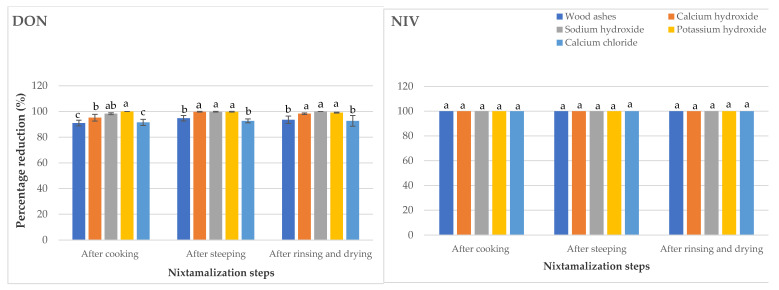

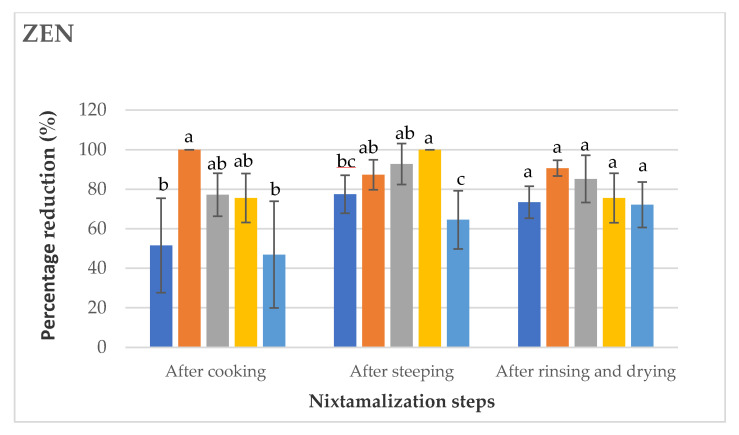
Reduction of other mycotoxins (DON, NIV, and ZEN) during the processing of nixtamalized maize samples using different nixtamalization cooking ingredients. Values are the means of five replicates ± standard error. Means followed by different letters are significantly different (*p* < 0.05) according to Tukey *post-hoc* test.

Cooking is a major thermal processing step during nixtamalization [12]. The results obtained after cooking showed that potassium hydroxide was the most effective cooking ingredient for the reduction of FB_1_, FB_2_, FB_3,_ and DON, while ZEN was most effectively reduced by calcium hydroxide treatment (Figure 1 and Figure 2). Park et al. [29] pinpointed potassium hydroxide as a feasible compound for alkaline hydrolysis of FBs. The concentration of ZEN found below the LOD in maize samples cooked with calcium hydroxide indicates that in addition to the isomerization of trans-ZEN to cis-ZEN in maize, this cooking ingredient attacked the lactone ring of ZEN, leading to the degradation of ZEN into an undetectable biotransformation product(s) [10].

For the steeping step, calcium chloride was found to be the least effective in reducing FB_1_ and ZEN. Moreover, calcium chloride in addition to wood ashes gave the least reduction for DON. This suggests that incomplete dehulling involved in the use of calcium chloride [11] led to a decrease in the reduction of fumonisins (FBs) content. The research outcome is consistent with Fandohan [30] who observed that removal of pericarp, referred to as dehulling, brought about a reduction in FBs concentrations. The ineffectiveness of wood ashes for the reduction of DON, with respect to other alkaline cooking ingredients, supports the point made by Santiago et al. [12] that wood ashes mainly consist of calcium carbonate with a lower alkaline pH value as compared to calcium hydroxide, sodium hydroxide, or potassium hydroxide. All cooking ingredients tested gave similar reduction results for FB_2_; however, the alkaline cooking ingredients gave the highest FB_3_ reduction levels in maize. To an extent, this agrees with Schaarschmidt and Fauhl-Hassek [10] findings where it was reported that alkaline cooking allows the leaching of FBs into the water during maize steeping. In addition, according to Santiago et al. [12], wood ashes contain traces of calcium hydroxide which, in a way, explains the non-significant difference in the potential of wood ashes and calcium hydroxide to reduce FB_3_. In the final *nixtamal* (after rinsing and drying), calcium chloride had the least reducing effect on all *Fusarium* mycotoxins in maize but there was no statistically significant difference in the ability of all the tested nixtamalization cooking ingredients to reduce ZEN. Ryu et al. [31] earlier noted that the use of chemical treatments for ZEN reduction in foods has not recorded any desired result.

As shown in Figure 1 and in line with Schaarschmidt and Fauhl-Hassek [10], the incomplete mycotoxins reduction recorded after rinsing and drying step in some instances (wood ashes and calcium chloride—FB_2_; wood ashes, potassium hydroxide and calcium chloride—FB_3_) may be due to the release of mycotoxins from matrix components of maize, which depends on the processing condition. This also explains the decreased percentage reduction for ZEN in the final nixtamal following potassium hydroxide treatment after rinsing and drying when compared to that obtained after steeping (Figure 2).

Although the accumulation of hydrolyzed and biotransformed products was not investigated in this study, it is important to note that there is a possibility of the presence of modified mycotoxins in the final nixtamal as a result of the solubility, changes in the structure, and molecular masses of the parent mycotoxins [32]. According to these authors, i.e., Freire and Sant’Ana [32], these modified mycotoxins produced via processing may be toxic to human health.

### 2.2. Influence of Different Nixtamalization Cooking Ingredients on Fusarium Mycotoxins during Nixtamalization of Sorghum

Assessment of the influence of the selected cooking ingredients on *Fusarium* mycotoxins (FB_1_, FB_2,_ and FB_3_; Table 3) contents during sorghum nixtamalization process was performed using artificially contaminated sorghum samples. Results showing the effect of washing on the reduction of target mycotoxins in the fungal-inoculated sorghum samples are highlighted in Table 3.

**Figure 3:**
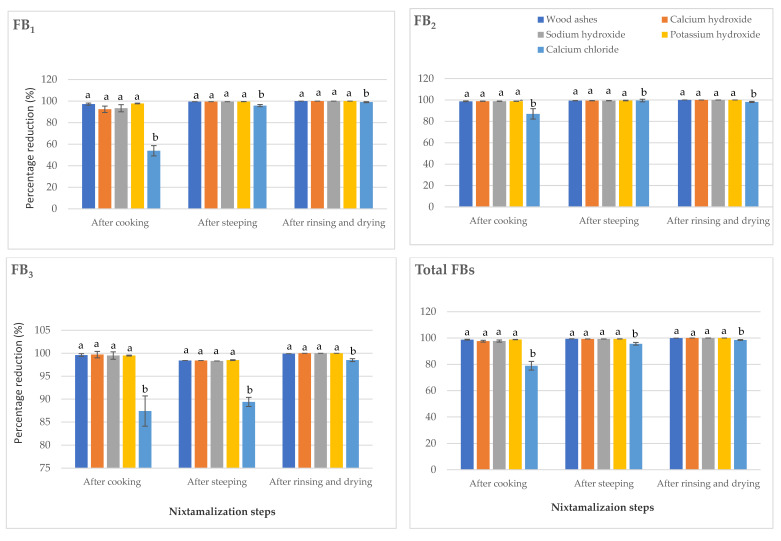
Reduction of fumonisins during the processing of nixtamalized sorghum samples using different nixtamalization cooking ingredients. Values are the means of five replicates ± standard error. Means followed by different letters are significantly different (*p* < 0.05) according to Tukey *post-hoc* test.

The reduced levels of these FBs after washing indicate that they are water-soluble and are easily washed away from the outer layer of the sorghum grains [23]. Schaarschmidt and Fauhl-Hassek [10] also reported that FBs are soluble and prone to leaching from grains into steeping and cooking solutions. Interestingly, there was no statistically significant difference (*p* > 0.05) in the results obtained from the effect of wood ashes, calcium hydroxide, sodium hydroxide, and potassium hydroxide during steeping, rinsing, and cooking stages on the reduction of FBs in sorghum. Calcium chloride, however, had the least potential for the reduction of these toxins (Figure 3).

As earlier mentioned, the use of calcium chloride involves partial dehulling [11], which affects its effectiveness for mycotoxins reduction in food when compared to other alkaline cooking ingredients that ensure better pericarp removal [17]. As discovered in this study, higher levels of FBs’ reduction were recorded in the nixtamalized sorghum compared to those of maize. These observations may be attributed to the absence of a waxy layer in sorghum grain that allowed the rapid uptake of water [33].

### 2.3. Effect of Different Nixtamalization Cooking Ingredients on the pH Values of Obtained Nejayote and Nixtamal

**Figure 4:**
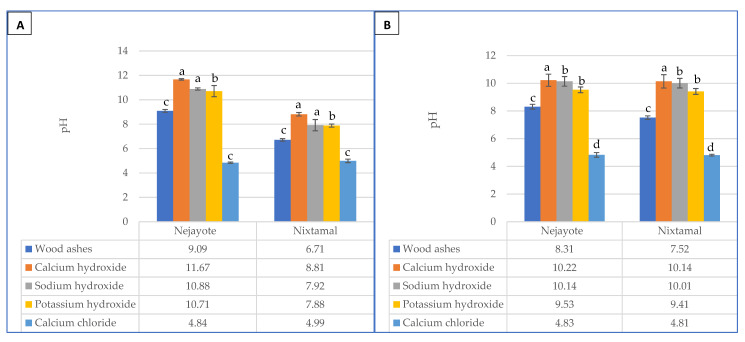
*Nejayote* and *nixtamal* pH values [(**A**): Maize; (**B**): Sorghum]. Values are the means of five replicates ± standard error. Means followed by different letters are significantly different (*p* < 0.05) according to Tukey *post-hoc* test.

Disposal of highly alkaline wastewater, *nejayote* (pH~ 9–12) remains a huge concern for food industries that process food by nixtamalization [34]. The *nejayote* and *nixtamal* from maize after calcium chloride treatment were found to have lower pH values (4.84 and 4.99, respectively), which were significantly different (*p* < 0.05) from those in the samples (pH range: 9.09–11.67 to 6.71–8.81) obtained via other cooking ingredients (Figure 4A). This is somewhat similar to the research findings of Pappa et al. [35] where the *nejayote* obtained from lime had a pH of 12. It can, thus, be seen that calcium chloride, a form of ecological nixtamalization, contributes minimally to environmental pollution when compared to other tested nixtamalization cooking ingredients [11,12].

Calcium chloride treatment also gave the lowest pH values in sorghum *negayote* (pH 4.83) and *nixtamal* (pH 4.81) samples. Interestingly, pH values of the sorghum *nejayote* and *nixtamal* samples after treatment with the different cooking ingredients followed a similar pattern recorded for maize, i.e., calcium hydroxide > sodium hydroxide > potassium hydroxide > wood ashes > calcium chloride (Figure 4B). This suggests that in comparison to other cooking ingredients, the use of calcium chloride for the production of sorghum *nixtamal* would raise the least concerns with respect to the problem of environmental pollution [11].

### 2.4. Principal Component Analysis of the Maize and Sorghum Data

**Figure 5:**
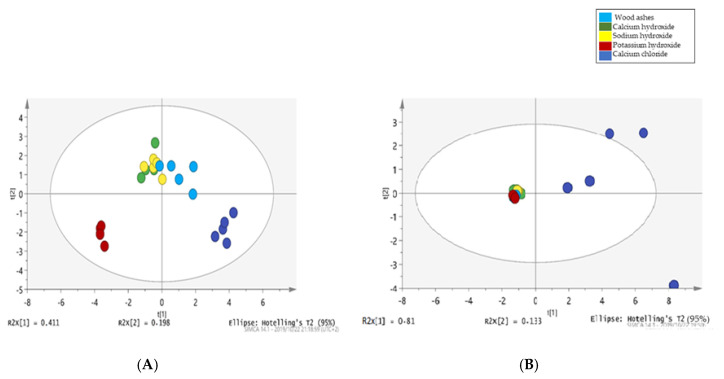
Investigational data analysis with unsupervised chemometric method, principal component analysis of: (**A**) Maize samples, and (**B**) Sorghum samples.

Principal component analysis (PCA) was performed to assess the association between the nixtamalization cooking ingredients and their *Fusarium* mycotoxins reduction patterns during nixtamalization. The unsupervised clustering method was followed to scrutinize the data structure of the percentage reduction of each *Fusarium* mycotoxin during the nixtamalization process, establish similarities among different cooking ingredients, and examine the presence or absence of outliers. Two principal components (PCs), i.e., PC1 and PC2, describing approximately 61% variation in the pareto-scaled data, were generated for the maize nixtamalization process (Figure 5A). PC1 explained 41.1% of the variance in the use of the different cooking ingredients for nixtamalization, while PC2 explained an additional 19.8% variation in the reduction of *Fusarium* mycotoxins when these cooking ingredients for nixtamalization were used. On the score plot (Figure 5A), a distinct separation of *nixtamal* from potassium hydroxide was observed in quadrant 3, while that produced from calcium chloride treatment was in the fourth quadrant.

In comparison with the clusters of potassium hydroxide and calcium chloride nixtamal, Figure 5A showed a somewhat close relationship between the clusters of the percentage mycotoxins reduction pattern of wood ashes, calcium hydroxide, and sodium hydroxide. The observed clear separation of some of the clusters reflects the differences in the chemical properties and reactions involved when different cooking ingredients for nixtamalization were used, which in turn led to notable changes in pH values.

The PCA scores plot of the nixtamalized sorghum samples is presented in Figure 5B. PC1 and PC2 account for 94.3% of the variation in the nixtamalized sorghum samples data with clear discrimination into two broad clusters. Mycotoxin reduction in the *nixtamal* cooked with calcium chloride was scattered toward the right, while the *nixtamal* obtained using other cooking ingredients clustered near the center position, which may be attributed to their similar chemical properties [12].

These clusters from wood ashes, calcium hydroxide, sodium hydroxide, and potassium hydroxide treatments as shown in Figure 5B, to a large extent, also reflect the earlier observed similar ability of these cooking ingredients to reduce FBs in sorghum. In contrast, the *nixtamal* cooked with calcium chloride showed high variation with major outliers. In line with Girolamo et al. [36], physico-chemical changes that take place in food during nixtamalization, like starch gelatinization, can lead to the release of matrix-associated FBs in the nixtamal. The degree of this gelatinization process depends on the breaking of hydrogen bonds of the hydroxyl groups present in the starch chains. Hence, the dissociation nature of compounds/ingredients used in nixtamalization may significantly affect starch gelatinization [12].

In the current study, calcium hydroxide, sodium hydroxide, and potassium hydroxide used as cooking ingredients for nixtamalization were dissociated into hydroxyl ion (OH^−^), which easily penetrated the starch granule, breaking the hydrogen bonds between water molecules and hydroxyl groups of the starch chains. This then led to an increase in the penetration power of water molecules and an enhanced gelatinization process in samples obtained from these cooking ingredients [12]. For calcium chloride, the gelatinization of starch granules is somewhat reduced as it dissociates into Cl^−^, which triggers the formation of hydrogen bonds between water molecules while resisting starch hydroxyl groups [17]. Generally, the difference in PCA plots for percentage mycotoxins reduction in maize and sorghum show that the mycotoxin reduction patterns in cereals are a function of their food matrices.

## 3. Conclusions

This study offers valuable information on the impact of five nixtamalization cooking ingredients on *Fusarium* mycotoxins reduction in maize and sorghum during nixtamalization. The experimental results revealed that in addition to FBs produced in maize and sorghum grains inoculated with *F. verticillioides* maintained under similar experimental conditions, other mycotoxins such as DON, NIV, and ZEN were also produced in the inoculated maize sample. Among the tested cooking ingredients for nixtamalization, calcium chloride was generally found to have the least reducing effect on the concentrations of the target mycotoxins in the final maize *nixtamal*. For sorghum, wood ashes, calcium hydroxide, sodium hydroxide, and potassium hydroxide were also more effective than calcium chloride in reducing FBs. Notwithstanding, the *nejayote* and *nixtamal* obtained from maize and sorghum treated with calcium chloride had the lowest pH values which are within the acidic range.

Overall, the research revealed that sodium hydroxide and potassium hydroxide can be used as alternative nixtamalization cooking ingredients to calcium hydroxide in reducing these FB analogs in contaminated maize and sorghum during nixtamalization. This further supports the use of sodium hydroxide for pre-cooking grains in pozole preparation as practiced in the nixtamalized maize flour industry. Although calcium chloride had the least significant effect on *Fusarium* mycotoxins reduction in the final *nixtamal* when compared with other cooking ingredients, it may still be used when there are environmental concerns regarding *nejayote* disposal. As the levels of parent mycotoxins in the final *nixtamal* obtained in this study were below the maximum allowable limit by the European Union, future studies are required to assess possible modified/bound/hidden mycotoxin products in the final *nixtamal,* aside from N-fatty acyl FB, arising from matrix-mycotoxin interaction during nixtamalization for potential identification and toxicity.

## 4. Materials and Method

### 4.1. Chemicals and Reagents

FB_1_, FB_2_, DON, deepoxy-deoxynivalenol (DOM), NIV, ZEN, and zearalanone (ZAN) standards were purchased from Sigma-Aldrich (Bornem, Belgium), while FB_3_ was from Promec Unit (Tynberg, South Africa). LC-MS grade methanol, glacial acetic acid, analytical-grade acetonitrile (Biosolve B. V., Valkenswaard, The Netherlands), analytical-grade methanol, ammonium acetate, formic acid, calcium hydroxide, calcium chloride, sodium hydroxide, potassium hydroxide (Merck, Darmstardt, Germany), dichloromethane, ethyl acetate (Acros Organics, Geel, Belgium), and n-hexane (VWR International, Zaventem, Belgium) were used including ultra-pure water from Arium^®^ pro Ultrapure Water System (Sartorius, Goettingen, Germany). Wood ashes were collected within Ghent environment, Belgium, while GracePure aminopropyl (NH_2_) solid-phase extraction (SPE) 1000 mg/6 mL cartridges were obtained from Grace Discovery Sciences (Lokeren, Belgium).

### 4.2. Preparation of the Growth Media and Inoculation of F. Verticillioides Strains

White maize and sorghum grains purchased at Bio Shop in Ghent, Belgium, were used for the experiment. Inoculation and incubation with *F. verticillioides* strains were achieved using solid potato dextrose agar (PDA) for 7 days at 25 °C in order to activate the strain. The maize and sorghum media were prepared by pouring 500 mL of distilled water in 2000 g of previously analyzed mycotoxin-free maize/sorghum grains. The media were vigorously shaken to avoid clumping, kept overnight, and sterilized in an autoclave for 15 min at 121 °C. The inoculation procedure as described by Shi et al. [7] and Medina et al. [37] was followed. This involved placing a piece of 4 mm diameter agar disc taken from the 7-day old colony of the strain grown on PDA at the center of the maize/sorghum media and incubating (25 °C) for 4 weeks. The media were harvested, dried at 40 °C until a constant weight was obtained, and kept cool prior to the nixtamalization experiment. Inoculated maize and sorghum samples were analyzed for their *Fusarium* mycotoxins content as described in Section 4.5 and Section 4.6 after the 4 weeks incubation.

### 4.3. Processing of Nixtamalized Maize and Sorghum

**Figure 6:**
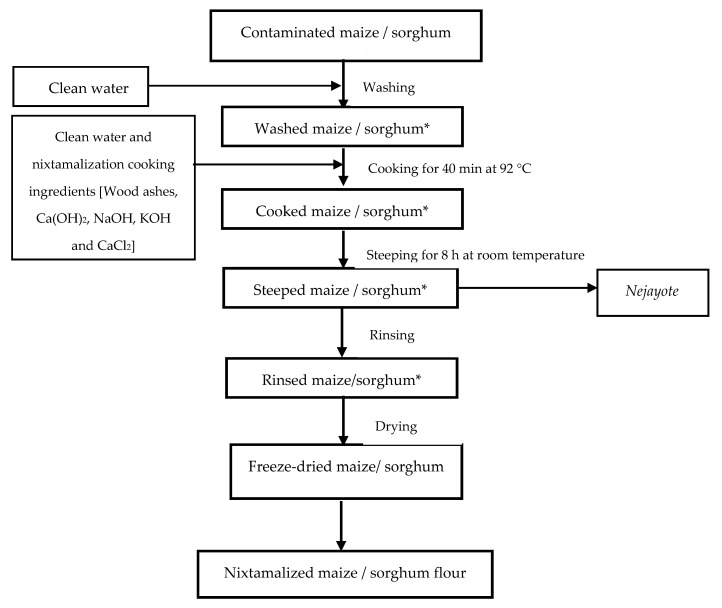
Schematic flow diagram of the nixtamalization of maize/sorghum using different nixtamalization cooking ingredients. (* Points where samples were taken for mycotoxins analysis).

The modified method of Villada et al. [38] was used for the nixtamalization process (Figure 6). Samples were prepared by first washing followed by cooking 100 g of toxigenic fungal-inoculated maize and sorghum grains with 200 mL purified water.

For the cooking process, contaminated maize and sorghum grains were treated with the different cooking ingredients for nixtamalization, i.e., wood ashes, calcium hydroxide, sodium hydroxide, potassium hydroxide, and calcium chloride (1 g each), by cooking at 92 °C for 40 min in 400 mL of water followed by steeping for 8 h at room temperature and discarding of the cooking liquor. The *nixtamal* was rinsed twice using purified water (3:1, *w/v*) for 60 s followed by freeze-drying using a Ruckwand VaCo 5 Standard freeze dryer (Zirbus Technology, Germany). Thereafter, the dried *nixtamal* as well as all other samples were milled to fine particle size (<200 µm) using an IKA M20 universal mill (Sigma-Aldrich, Bornem, Belgium), thoroughly homogenized, stored, and kept at −18 °C prior to analysis.

### 4.4. Preparation of Mycotoxin Standard Solution

Stock solutions of FB_1_, FB_2_, FB_3_, DON, NIV, ZEN, and ZAN were prepared in methanol at a concentration of 1 mg/mL. DOM (50 µg/mL) was obtained as a solution in acetonitrile. The working standard solutions were prepared by diluting the stock standard solutions in methanol and storing them immediately at −18 °C. The standard solution mixture of FB_1_ (5 µg/mL), FB_2_, FB_3_, DON (each 10 µg/mL), NIV (40 µg/mL), and ZEN (2.5 µg/mL) was then prepared from the working standard solutions.

### 4.5. Sample Extraction and Clean-Up

Extraction and clean-up of all the samples were performed following the procedure of Njumbe Ediage et al. [39]. Each sample (3 g) was spiked with internal standards [ZAN (2.5 µg/mL) and DOM (50 µg/mL)] and allowed to equilibrate in the dark for 15 min. Twenty milliliters of extraction solvent [methanol/ethyl acetate/water (70/20/10, *v/v/v*)] were added to each sample. The mixture was vortexed, agitated for 40 min using an overhead shaker (Agitelec, Paris, France), centrifuged for 10 min at 4000× *g*, and the supernatant transferred into a new centrifuge tube. Ten milliliters of n-hexane was added to the supernatant and defatting performed by agitation and centrifugation. The lower phase of the solution was subjected to solid-phase extraction (SPE) and the upper phase (n-hexane layer) discarded.

SPE involved purification using GracePure amino SPE cartridges. Due to the strong binding capability of the carboxylic acid functional groups of FBs to the resin of the amino SPE cartridges, the defatted extract was divided into two portions and subjected to different clean-up procedures. Each defatted extract (2.5 mL) was transferred to a centrifuge tube containing a 10 mL solution of dichloromethane/formic acid (95/5, *v/v*), vortexed, and centrifuged at 4000× *g* for 10 min. Thereafter, 12.5 mL part of the defatted extract was passed through an amino SPE (GracePure, 1000 mg) column, fixed on a vacuum elution manifold previously pre-conditioned with 10 mL of the extraction solvent. The eluate from the SPE was collected in a glass test tube. Both portions of the cleaned extracts in the dichloromethane/formic acid solution and after amino SPE clean-up were combined and evaporated at 40 °C to dryness using nitrogen gas at a gentle flow rate. The residue was reconstituted in 300 µL of mobile phase containing equal volumes of mobile phase A [water/methanol/acetic acid (94/5/1, *v/v/v*) + 5 mM ammonium acetate (0.385 g/L)] and mobile phase B [water/methanol/ acetic acid (2/97/1, *v/v/v*) + 5 mM ammonium acetate (0.385 g/L)] mixed with 200 µL of n-hexane. Prior to injection in the LC-MS/MS, the reconstituted extract was centrifuged for 10 min at 1000 g and further filtered using Ultra free PVDF centrifuge filters with a pore size of 0.22 µm (Millipore Bedford, MA, USA).

### 4.6. Liquid Chromatography-Tandem Mass Spectrometry

Identification and quantification of *Fusarium* mycotoxins were performed on a Waters Acquity UPLC apparatus paired to a Quattro premier XE Tandem Mass Spectrometer (Waters, Milford, MA, USA). The chromatographic conditions were similar to those described by Njumbe Ediage et al. [39] with a C18 column (150 mm × 2.1 mm i.d., 5 µm) preceded by a guard column (10 mm × 2.1 mm) of similar material (Waters, Zellik, Belgium). The analyte injection volume of 10 µL was used with mobile phases A and B set at a flow rate of 0.3 mL/min following a gradient elution program, and 28 min run time. The instrument was controlled, and data processed using the Masslynx version 4.1 and Quanlynx version 4.1 software (Manchester, UK). Limits of detection (LOD) and quantitation (LOQ) were established at 3.33 and 10 times the signal/noise ratio, respectively.

### 4.7. Statistical Analysis

The influence of different nixtamalization cooking ingredients on the reduction of *Fusarium* mycotoxins in maize and sorghum during nixtamalization was evaluated. An independent-sample *T*-test was performed where applicable while One-way analysis of variance (ANOVA) (SPSS^®^, v26, IBM Statistics for Windows, New York, NY, USA) of the results at 95% confidence level was carried out with Tukey’s test for the *post-hoc* analysis. In addition, Soft Independent Modelling of Class Analogy (SIMCA) software (version 14.1 Umetrics; Umea, Sweden) was used for the principal component analysis of the data obtained.

## Figures and Tables

**Table 1:** Estimated percentage recovery, limit of detection, and quantification (µg/kg) of *Fusarium* mycotoxins in maize and sorghum following LC-MS/MS analysis.

Mycotoxins	Maize LOD	Maize LOQ	Maize Recovery	Sorghum LOD	Sorghum LOQ	Sorghum Recovery
FB_1_	1.0	2.9	100.0	0.8	2.4	107.0
FB_2_	0.6	1.7	99.0	0.2	0.7	100.0
FB_3_	0.6	1.7	102.0	0.5	1.6	90.0
DON	3.1	9.2	102.0	N/A	N/A	N/A
NIV	3.5	10.6	99.0	N/A	N/A	N/A
ZEN	1.1	3.5	107.0	N/A	N/A	N/A

N/A: Not applicable; LOD: Limit of detection; LOQ: Limit of quantification; FB_1_: fumonisin B_1_; FB_2_: fumonisin B_2_; FB_3_: fumonisin B_3_; DON: deoxynivalenol; NIV: nivalenol; and ZEN: zearalenone.

**Table 2:** Concentrations of *Fusarium* mycotoxins in purchased maize, inoculated maize, and washed maize and estimated percentage reduction after washing following LC-MS/MS analysis.

*Fusarium* Mycotoxins	Purchased Maize (µg/kg)	Inoculated Maize (µg/kg)	Washed Maize (µg/kg)	Reduction (% R)
FB_1_	<LOD	2470.5 ^b^ ± 346.4	661.5 ^a^ ± 243.6	73.2
FB_2_	<LOD	604.7 ^b^ ± 84.9	204.6 ^a^ ± 79.7	66.0
FB_3_	<LOD	409.0 ^b^ ± 59.5	162.9 ^a^ ± 24.8	60.0
Total FBs	<LOD	3884.3 ^b^ ± 482.0	1029.0 ^a^ ± 344.3	73.5
DON	<LOD	330.9 ^b^ ± 17.6	224.3 ^a^ ± 67.9	32.3
NIV	<LOD	104.9 ± 15.5	<LOD	100.0
ZEN	<LOD	15.9 ^a^ ± 2.5	15.0 ^a^ ± 4.9	6.2

Values are the means of five replicates ± standard error. Means followed by different letters are significantly different (*p* < 0.05). LOD: Limit of detection; FB_1_: fumonisin B_1_; FB_2_: fumonisin B_2_; FB_3_: fumonisin B_3_; Total FBs: FB_1_ + FB_2_ + FB_3_; DON: deoxynivalenol; NIV: nivalenol; and ZEN: zearalenone.

**Table 3:** Concentrations of *Fusarium* mycotoxins in purchased sorghum, inoculated sorghum, washed sorghum, and estimated percentage reduction after washing following LC-MS/MS analysis.

*Fusarium* Mycotoxins	Purchased Sorghum (µg/kg)	Inoculated Sorghum (µg/kg)	Washed Sorghum (µg/kg)	Reduction (% R)
FB_1_	<LOD	816.7 ^b^ ± 346	416.5 ^a^ ± 244	49.0
FB_2_	<LOD	1079.3 ^b^ ± 134.3	362 ^a^ ± 159.6	66.4
FB_3_	<LOD	1364.8 ^b^ ± 196.8	563.3 ^a^ ± 212.5	58.7
Total FBs	<LOD	3260.9 ^b^ ± 397.3	1341.8 ^a^ ± 365.3	58.8

Values are the means of five replicates ± standard error. Means followed by different letters are significantly different (*p* < 0.05). LOD: Limit of detection; FB_1_: fumonisin B_1_; FB_2_: fumonisin B_2_; FB_3_: fumonisin B_3_; and Total FBs: FB_1_ + FB_2_ + FB_3_.

## Data Availability

The data presented in this study are available on request from the corresponding authors.
